# Generalized bone loss in early rheumatoid arthritis patients followed for ten years in the biologic treatment era

**DOI:** 10.1186/1471-2474-15-289

**Published:** 2014-09-02

**Authors:** Glenn Haugeberg, Knut Bjørn Helgetveit, Øystein Førre, Torhild Garen, Hege Sommerseth, Anne Prøven

**Affiliations:** Department of Rheumatology, Hospital of Southern Norway Trust, Servicebox 416, Kristiansand, 4632 Norway; Department of Neuroscience, Division of Rheumatology, Norwegian University of Science and Technology, Trondheim, Norway; Department of Rheumatology, Martina Hansens Hospital, Bærum, Norway; Department of Rheumatology, Oslo University Hospital, Oslo, Rikshospitalet Norway

## Abstract

**Background:**

Osteoporosis is a well-known extra articular manifestation in rheumatoid arthritis (RA). Biologic disease modifying anti rheumatic drugs (DMARDs) has been shown to be superior to synthetic DMARDs to reduce bone destruction including generalized bone loss in RA. Our aim was to study short- and long term changes in hip and spine bone mineral density (BMD) in early RA patients treated during the first decade with available biologic DMARDs.

**Methods:**

RA patients diagnosed at an out-patient clinic between 1999 and 2001 were consecutively enrolled. Demographic, disease and treatment data were collected and BMD was assessed by dual energy X-ray absorptiometry at baseline and after 2, 5 and 10 years.

**Results:**

The 92 included RA patients had a baseline mean age (SD) of 50.9 (13.3) years and symptom duration of 12.4 (6.7) months, 62.0% were women and 66.3% were RF positive. In the first 2 years ever use of biologic DMARDs was 18.5%, synthetic DMARDs 91.3% and prednisolone 62.0% whereas the figures for the subsequent 8 years were 62.6%, 89.2% and 51.4%, respectively. The annual rate of BMD loss in the first 2 years and the subsequent 8 years was at femoral neck −1.00% vs. −0.56%, at total hip −0.96% vs. −0.41% and at spine L1−4 -0.42% vs. 0.00%.

**Conclusions:**

Our study adds evidence that aggressive anti-inflammatory treatment including biologic DMARDs reduces the rate of bone loss in RA. Indicating that the burden of osteoporosis is reduced in RA patients treated in clinical practice in the new millennium.

**Electronic supplementary material:**

The online version of this article (doi:10.1186/1471-2474-15-289) contains supplementary material, which is available to authorized users.

## Background

Osteoporosis and its clinical consequence fracture is a well-known extra articular manifestation in rheumatoid arthritis (RA). In both male and female RA populations the prevalence of reduced bone density has been reported to be doubled compared to the background population [1, 2]. Patients with RA are at increased risk for both vertebral [3] and non-vertebral fractures [4]. Generalized bone loss assessed at hip or spine occurs early in the disease [5] and is related to inflammatory activity [6].

During the last 10–15 years the importance of treating RA patients towards remission or low disease activity using outcome measures has been well documented and become the recommended treatment strategy [7]. In the same time period new potent anti-inflammatory drugs the biologic disease modifying anti-rheumatic drugs (DMARDs) has become available for clinical use [8]. Treatment with biologic DMARDs, e.g. tumor necrosis factor alpha (TNF) inhibitors has been shown not only to reduce the development of erosion but also to reduce the rate of generalized bone loss in RA [9–11]. Studies have been performed to examine bone loss in RA [5, 6, 9–14]. These studies however are limited by their rather short observational period. Furthermore there is a lack of long term follow up studies following patients from the early phase of the disease.

Thus the primary aim of the present study was to examine short term and long term changes in bone density at hip and lumbar spine in patients with early RA treated during the last decade. Second to search for predictors and associates with change in bone density.

## Methods

### Patients, disease measures and treatment

In an out-patient rheumatology clinic between 1999 and 2001 patients diagnosed with RA were consecutively included in a prospective observational follow up study. The patients had to fulfil the American College of Rheumatology 1987 revised classification criteria for RA [15]. According to protocol collection of demographic, clinical and treatment data was done at inclusion and after 6 months, 2, 5 and 10 years follow up. At all visits, data for demographic variables, disease characteristics, disease activity and health status were collected either by interview, clinical assessment, questionnaires or by reviewing the medical records. Disease activity was assessed by c-reactive protein (CRP), erythrocyte sedimentation rate (ESR) and the composite disease activity score (DAS) calculated from 28 swollen and 28 tender joint count and ESR (DAS28ESR3). We used DAS28ESR3 because data on patient’s global assessment used for DAS calculation was not collected. We also registered data for rheumatoid factor (RF) at baseline and anti-cyclic citrullinated peptide (anti-CCP) during follow up. Modified Health Assessment Questionnaire (MHAQ) was used for assessment of physical function [16].

At all visits the use of prednisolone and synthetic and biologic DMARDs were registered. Furthermore treatment information in-between the visits were collected based on information in the medical records which also included the use of intra-muscular and intra-articular glucocorticosteroids (GC) and osteoporosis treatment. Cumulative doses of GC were calculated and transformed into prednisolone equivalent doses.

At 10 years visit serum 25-hydroxyvitamin D (s-25(OH) D) was measured. Severe vitamin D deficiency was defined as s-25(OH) D levels lower than 12.5 nmol/l and moderate deficiency as 12.5-25 nmol/l [17].

### Bone density

Bone mineral density (BMD) was assessed at baseline, 2, 5 and 10 years follow up using the same dual energy X-ray absorptiometry (DXA) equipment (Lunar Prodogy). Trained osteoporosis nurses performed all standardized BMD measurements at hip (femoral neck and total hip) and lumbar spine L1-4. Left hip was measured. If left hip could not be measured the right hip was measured. The DXA machine was stable for the whole period. Long term spine phantom precision expressed as coefficient of variation (CV) was 0.68%. In vivo-short term precision based on duplicate measurement of 30 individuals was for femoral neck left side 1.93% and right side 1.80%, for total hip left side 0.79% and right side 0.75% and for spine L1-4 0.88%.

### Analysis and statistical tests

Due to a delay in installing the DXA machine at the hospital prior to study start, BMD in the first 42 included RA patients could not be measured at baseline. Missing baseline BMD values was calculated from the patient’s 2 years BMD value and adjusted for the mean percentage change calculated from all patients with available baseline and 2 years BMD data. In patients with missing DXA data between two measurement time points missing BMD was calculated using the annual percentage change adjusted for the time period. The BMD data were analyzed with the available data and with imputation of missing data separately.

For descriptive statistics continuous variables with normal distribution was presented as mean with standard deviation (SD) or with 95% confidence interval (CI) whereas variables with non-normal distribution also were presented with median and interquartile range [IQR]. Categorical variables were presented as numbers and percentage.

Percentage BMD change between DXA measurement periods was calculated.

For group comparison, we used t-test for continuous variables if normal distributed and Mann–Whitney U test if not normal distributed and chi-square test for categorical variables.

To explore for predictors and associates with change in BMD for the period 0–2 years and 2–10 years, we used unadjusted and adjusted linear regression analysis. For the multivariate linear regression models we used forward procedure. Included were variables with a p value ≤0.1 tested in univariate linear regression.

Statistical tests were performed using PASW Statistics 18 (IBM SPSS statistics). Significance level was p < 0.05.

### Ethics and legal aspects

The study was approved by the regional committee for ethics and medical research, health region II in Norway (REK-S-98093). All patients gave written informed consent before inclusion.

## Results

### Patients, disease measures and treatment

A total of 94 RA patients were included. Two patients had only baseline DXA values and were excluded from the analysis. Among the 92 patients (35 men and 57 women) 91 were Caucasian and 1 was Asian. Baseline patient characteristics for demographic, disease measures, disease activity and health status are shown in Table 1. For variables listed in Table 1 a statistical significant difference between men and women was only shown for age (mean 55.5 vs. 48.0 years, p = 0.008), weight (mean 84.3 vs. 68.5 kg, p < 0.001), height (mean 1.78 vs. 1.63 m, p < 0.001) and CRP (mean 40.1 vs. 21.9 mg/dl, p = 0.014, median 27.5 vs. 13.0, p = 0.025).

**Table 1:** **Baseline characteristics in 92 early rheumatoid arthritis patients**

Patient data	
Age (years)	50.9 (13.3)
Women	57 (62.0)
Females in menopause (n = 51)*	36 (70.6)
Weight (kg)	74.5 (16.9)
Height (m)	1.69 (0.10)
BMI (kg/m2)	25.8 (4.4)
Current smoking	35 (38.0)
Disease data	
Symptom/disease duration (months)	12.4 (6.7)
Anti-CCP positive, (n = 82)** ^†^	54 (65.9)
High titer CCP positive >3X^‡^ (n = 82)**	52 (63.4)
RF positive, (n = 86)*	57 (66.3)
High titer RF positive >3X^‡^ (n = 86)**	46 (53.5)
ESR (mm/hr)	29.6 (21.2)
	24.5 [21.0]
CRP (mg/dl) (n = 90)**	28.8 (34.4)
	17.5 [27.3]
DAS28ESR3	5.2 (1.1)
MHAQ (0–3) (n = 91)**	0.68 (0.51)
	0.63 [0.75]

Continuous variables with normal distribution are presented as mean with standard deviation (SD) whereas variables with non-normal distribution are also presented as median with interquartile range [IQR]. Categorical variables are presented as numbers and percentage (%).

*Varies from 57 due to missing values.

**Varies from 92 due to missing values.

^†^Anti-CCP data are the first available values in medical records.

^‡^Values three times higher than upper limit of normal values for the test.

*BMI*: Body mass index; *Anti-CCP*: Anti cyclic citrullinated peptide; *RF*: Rheumatoid factor; *ESR*: Erythrocyte sedimentation rate; *CRP*: C-reactive protein; *DAS28ESR3*: Disease activity score based on 28 joint count (swollen and tender joints) and ESR; *MHAQ*: Modified health assessment questionnaire.

In Table 2 is shown mean values for ESR, CRP, DAS28ESR3 and MHAQ and the use of biologic and synthetic DMARDs and GC for the 0–2 years and 2–10 years period.

**Table 2:** **Disease measures and treatment in 92 early rheumatoid arthritis patients followed for 10 years**

	Period 0–2 years (n = 92)	Period 2–10 years (n = 74)	P value
Mean ESR (mm/hr)	21.2 (12.0)^†^	13.2 (6.8)^‡^	<0.001
Mean CRP (mg/dl)	18.8 (14.9)^†^	8.1 (7.0)^‡^	<0.001
	14.7 [18.0]	5.5 [8.6]	
Mean DAS28ESR3	4.17 (0.88)^†^	2.92 (0.82)^‡^	<0.001
Mean MHAQ (0–3)	0.42 (0.30)^†^	0.27 (0.27)^‡^	<0.001
	0.33 [0.45]	0.18 [0.42]	
Ever users synthetic DMARDs	84 (91.3)	66 (89.2)	<0.001
Ever users methotrexate	71 (77.2)	61 (82.4)	<0.001
Ever users biologic DMARDs	17 (18.5)*	46 (62.6)**	<0.001
Ever users prednisolone	57 (62.0)	38 (51.4)	<0.001
Ever users any GC***	76 (82.6)	59 (79.7)	0.400
Mean prednisolone equivalent dose (gram)****	4.56 (4.71)	7.29 (10.53)	0.012
	2.77 [7.50]	2.09 [11.53]	

Continuous variables with normal distribution are presented as mean with standard deviation (SD) whereas variables with non-normal distribution also are presented as median with interquartile range [IQR]. Categorical variables are presented as numbers and percentage (%).

^†^Calculated from baseline, 6 months and 2 years data.

^‡^Calculated from 2 years 5 years and 10 years data.

*All treated with tumor necrosis factor inhibitors.

**All treated with tumor necrosis factor inhibitors, 6 patients also had been treated with other biologic DMARDs (rituximab and/or tocilizumab).

***Includes prednisolone, intraarticular and intramuscular GC injections.

****The cumulative doses of any GC given were calculated and transformed into prednisolone equivalent doses.

*ESR*: Erythrocyte sedimentation rate; *CRP*: C-reactive protein; *DAS28ESR3*: Disease activity score based on 28 joint count (swollen and tender joints) and ESR MHAQ: Modified health assessment questionnaire; *DMARDs*: Disease modifying anti-rheumatic drugs; *GC*: Glucocorticosteroids.

The ever use of biologic DMARDs increased from 18.5% in the period 0–2 years to 62.6% in the period 2–10 years. All patients treated with biologic DMARDs had been using TNF inhibitors. The median dose among users of prednisolone was 5.0 mg at all follow up visits with a range of 2.5 mg to 15.0 mg. No statistically significant differences were seen between men, pre- and post-menopausal women on the use of synthetic DMARDs, biologic DMARDs, prednisolone, any GC use or cumulative GC dose for the two first years and the subsequent eight years of follow up.

During follow up minor differences between men and women and between pre- and post-menopausal women was found for disease measures and treatment listed in Table 2.

During follow up 16.3% (5 men and 10 women) were using anti-resportive osteoporosis treatment (ART), 4 oestrogen and 11 bisphosphonates and 70.7% (23 men and 42 women) were using calcium and/or vitamin D.

At 10 years visit 93.2% (69 out of 74 patients) had vitamin D measured. Mean (SD) value for s-25(OH) D was 72.9 (26.1) nmol/l. None had severe vitamin D deficiency (<12.5 nmol/l) and only two patients had moderate vitamin D deficiency (12.5-25.0 nmol/l).

### Bone density

**Figure 1:**
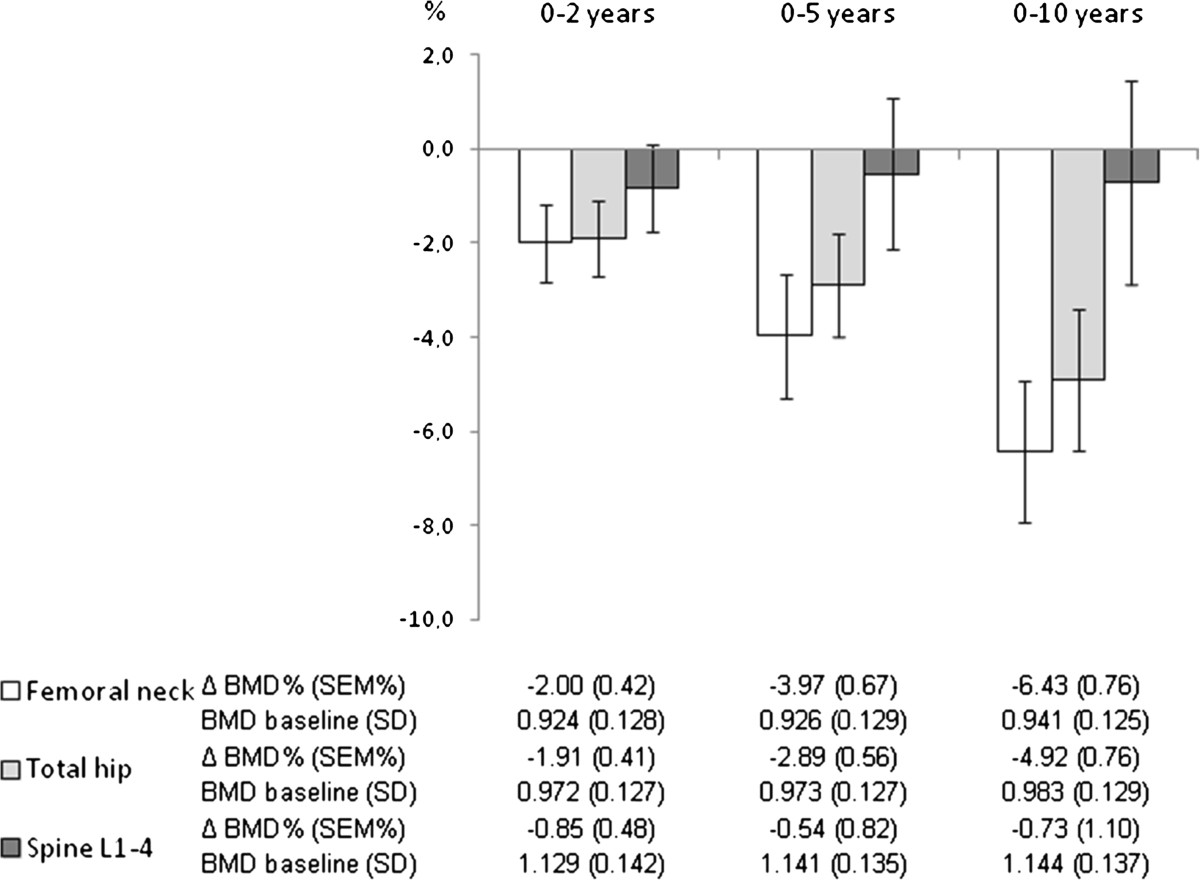
**Percentage change (mean with 95% confidence interval) in bone mineral density (BMD) at femoral neck, total hip and spine L1-4 after 2, 5 and 10 years follow up in early rheumatoid arthritis patients.** Numbers below the graph shows the mean percentage BMD change with standard error of mean (SEM) for the period and baseline BMD values (g/cm2) with standard deviation (SD) for patients with follow up data.

Among the 92 included patients 74 (80.4%) patients had their last DXA performed at 10 years, 14 (15.2%) patients at 5 years and 4 (4.3%) patients at 2 years follow up. Apart from missing values at baseline (42 patients) as described in material and method section 3 patients did not have DXA measurement performed at 2 years follow up and 6 patients at 5 years follow up. In 7 patients right hip instead of left hip was measured.As shown in Figure 1 a significant bone loss (p < 0.001) was found between baseline and 2, 5 and 10 years follow up at femoral neck and total hip but not at lumbar spine.

**Figure 2:**
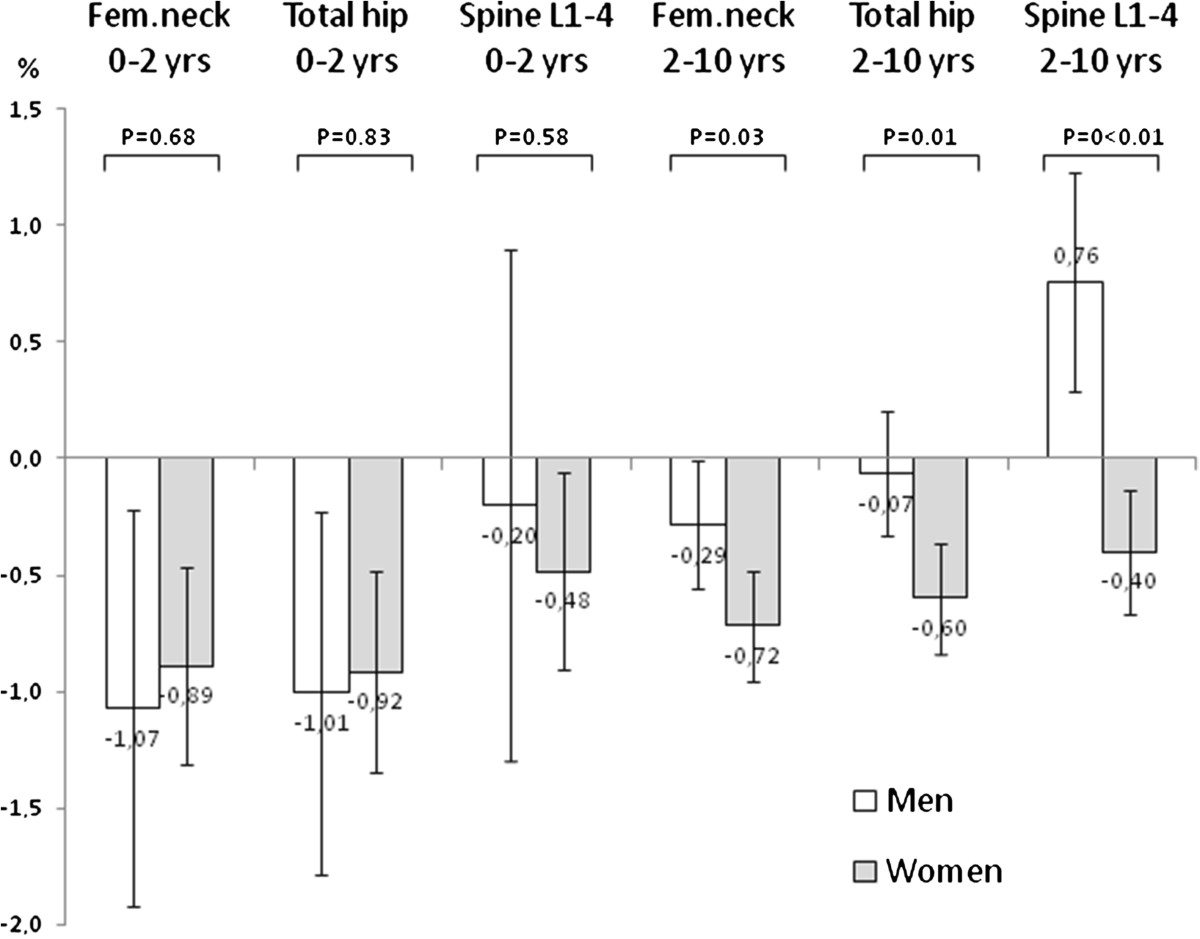
**Annual change (mean percentage with 95% confidence intervals of mean) in bone mineral density (BMD) at femoral neck (fem.neck), total hip and spine L1-4 for the first 2 years period and for the subsequent 8 years period in men and women with early rheumatoid arthritis followed for up to 10 years.**

The annual rate of bone loss for the period’s baseline to 2 year, 2 to 5 years, 5 to 10 years and 2 to 10 years follow up was at femoral neck −1.00%, −0.68%, −0.54% and −0.56% and at total hip −0.96%, −0.39%, −0.46% and −0.41%, and at spine L1-4 −0.42%, +0.04%, −0.09% and 0.0%, respectively. The annual rate of bone loss in the first 2 years was significantly higher in patients treated with biologic DMARDs compared with non biologic DMARDs treated patients at femoral neck (−2.07% vs −0.74%, p = 0.019) and total hip (−1.92 vs. −0.76%, p = 0.034) but not at lumbar spine L1-4 (−1.27% vs. −0.21%, p = 0.10). No significant differences in bone loss was found between the biologic and non biologic DMARD treated patients neither for the 2–5 years period (femoral neck −0.87% vs. −0.52%, total hip −0.57% vs. -0.28%, spine L1-4 −0.39% vs. 0.50%), the 5–10 years period (femoral neck −0.46% vs. −0.64%, total hip −0.41% vs. -0.23%, spine L1-4 −0.02% vs. −0.17%) or for the whole 2–10 years period (femoral neck −0.56% vs. −0.56%, total hip −0.32% vs. −0.49%, spine L1-4 −0.13% vs. 0.19%). When the data were analyzed comparing patients who were treated with biologic DMARDs and/or prednisolone with patients who were not treated with these drugs the same overall pattern was seen (data not shown).As shown in Figure 2 a statistical significant difference in annual percentage bone loss between men and women was seen for the 2–10 years but not for the 0–2 years period.

**Figure 3:**
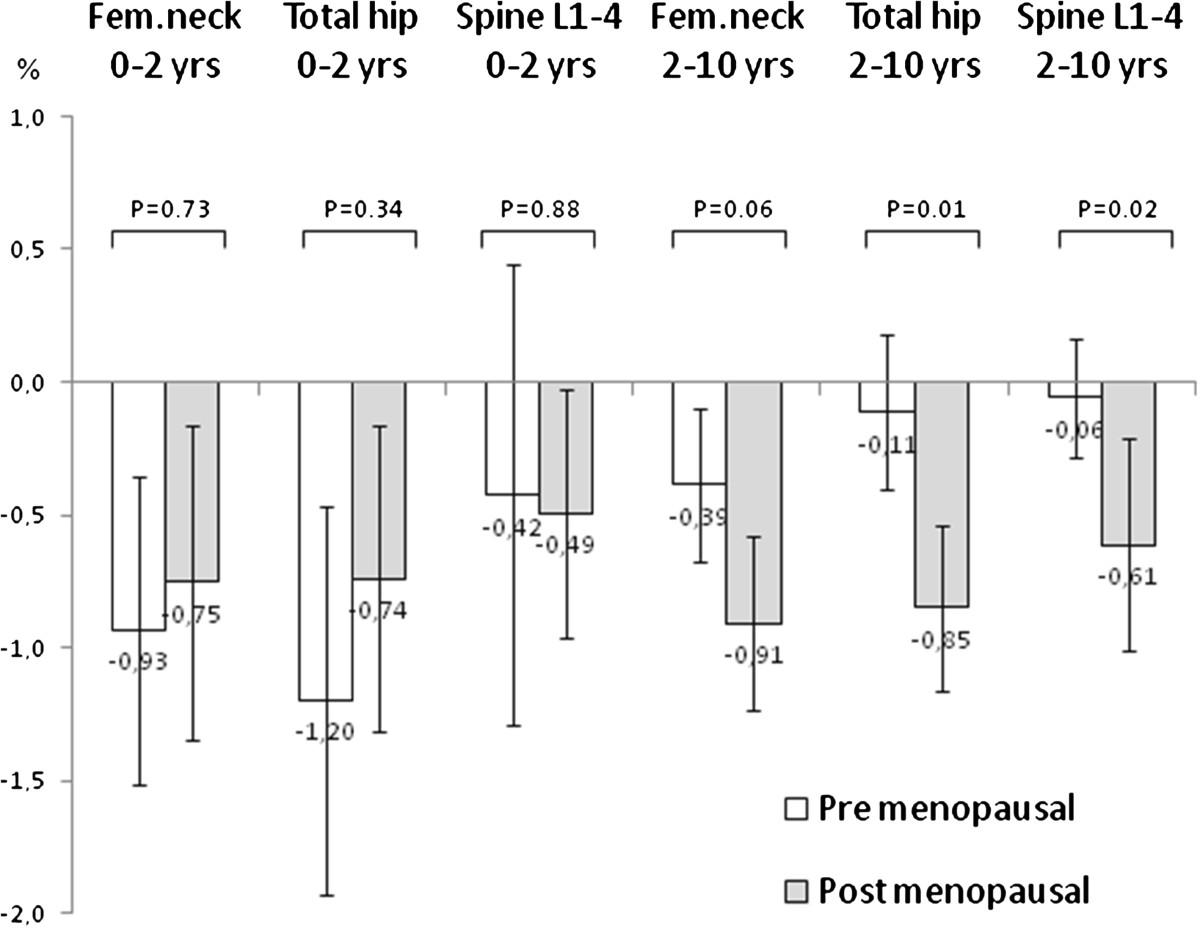
**Annual change (mean percentage with 95% confidence intervals of mean) in bone mineral density (BMD) at femoral neck (fem.neck), total hip and spine L1-4 for the first 2 years period and for the subsequent 8 years period in women with pre- and post-menopausal status with early rheumatoid arthritis followed for 10 years.**

In 8 patients (7 men and 1 woman) in the period 2–10 years annual gain in spine L1-4 BMD exceeded one percent (range 1.03% − 4.89%). Mean age in these eight patients was 54.1 (8.8) years. On the DXA spine scans from these patients areas with increase of more dense bone and signs of new bone formation present as osteophytes were visible explaining the increase in bone density.As shown in Figure 3 a difference in annual rate of bone loss between pre- and post-menopausal women was seen for the period 2–10 years but not for the period 0–2 years.

No statistically significant differences in BMD change were seen between men and pre-menopausal women for the 0–2 years and the 2–10 years period apart from annual change in bone density at spine L1-4 for the 2–10 years period. Between men and post-menopausal women a statistically significant difference in change in bone density was seen at hip and spine for the 2–10 years period but not for the 0–2 year period.

No statistically significant difference in bone change during follow up was seen either between never and ever users of ART.

### Validity of BMD results using imputation of missing data

Between patients with and without DXA baseline values no significant difference was seen for BMD at femoral neck, total hip and spine L1-4 at 2, 5 and 10 years follow up, or for changes in bone density between 2 and 5, 2 and 10 and 5 and 10 years follow up. Further no statistically significant difference was seen between the two groups for variables listed in Tables 1 and 2, this both at baseline, at 2 years follow up and in-between.When only patients with available data at all visits were analyzed the same magnitude and pattern of bone loss was seen compared to when data were handled with imputation of missing data as shown in Figure 1. The mean BMD change for baseline-2 years, baseline-5 years and baseline-10 years follow up was at femoral neck (n = 36) −1.75%, −4.61%, −6.83% at total hip (n = 36) −1.07%, −3.20%, −4.81% and at spine L1-4 (n = 28) −0.54%, −1.33% and −1.55%, respectively.

### Fractures

One patient had an ankle and a femur fracture and two patients had clinical costa fracture during follow up.

### Associates with change in bone density during follow up

The baseline variables listed in Table 1 and mean values of disease measures (ESR, CRP, DAS28ESR3 and MHAQ,) and treatment (ART, DMARDs, biologic DMARDs and GC) during follow up listed in Table 2 was one by one tested for their association with bone loss for the first 0–2 year period and for the 2–10 year period.

For the baseline disease measures ESR, CRP, DAS28ESR3 and MHAQ a statistically significant or a border significant association with bone loss was only found for the 0–2 year period for MHAQ (B-0.015 P = 0.047) and DAS28ESR3 (B-0.007, P = 0.051) at femoral neck and for ESR (B 0.000 P = 0.050), DAS28ESR3 (B −0.018 P < 0.001) and MHAQ (B-0.030 P = 0.004) at spine L1-4.

In Table 3 variables with an association with BMD change (p value ≤ 0.1) for the 0–2 year period and/or the 2–10 years period at femoral neck and/or total hip and/or spine are shown.

**Table 3:** **Associates with bone loss in 92 rheumatoid arthritis patients followed for 10 years**

	Change in bone mineral density					
	Femoral neck		Total hip		Spine L1-4	
	0-2 yrs	2-10 yrs	0-2 yrs	2-10 yrs	0-2 yrs	2-10 yrs
	Beta	Beta	Beta	Beta	Beta	Beta
	p value	p value	p value	p value	p value	p value
Age (yrs)	-	-	-	-	-	B 0.244 P = 0.048
Women (N/Y)	-	B-0.029 P = 0.027	-	B-0.039 P = 0.004	-	B-0.104 P = 0.000
Menopause (N/Y)	-	B-0.033 P = 0.049	B 0.016 P = 0.076	B-0.053 P = 0.003	-	B-0.051 P = 0.063
Smoking (N/Y)	-	B-0.028 p = 0.036	-	B-0.024 P = 0.080	-	-
RF (N/Y)	-	B-0.030 P = 0.030	-	B-0.029 P = 0.047	-	B-0.053 P = 0.044
MHAQ (0–3)	B −0.027 p = 0.034	-	-	-	-	-
ESR (mm/hr)	-	-	-	-	B-0.189 P = 0.092	
DAS28ESR3	B −0.010 P = 0.030	B-0.015 P = 0.057	-	B-0.016 P = 0.055	B-0.013 P = 0.037	-
Ever use of biologic DMARDs (N/Y)	B-0.023 P = 0.024	-	B-0.019 P = 0.058	-	B-0.023 P = 0.091	B-0.052 P = 0.039
Cumulative equivalent prednisolon (gram)	B-0.002 P = 0.024	-	B-0.003 P = 0.003	-	-	-

Variables are tested for their association with change in hip and spine bone density for the follow up periods 0–2 years and 2–10 years in early rheumatoid arthritis patients using univariate linear regression analysis. Unstandardized Beta values are only shown for variables with a p value ≤ 0.1.

*Yrs*: Years; *N/Y*: No/yes; *RF*: Rheumatoid factor; *MHAQ*: Modified health assessment questionnaire; *ESR*: Erythrocyte sedimentation rate; *DAS28ESR3*: Disease activity score based on 28 joint count (swollen and tender joints) and ESR; *DMARDs*: Disease modifying anti-rheumatic drugs.

In the multivariable models with forward procedure variable independently associated with loss in BMD for the 0–2 years period was for femoral neck ever use of biologic DMARDs (B −0.023, p = 0.024), for total hip cumulative dose of GC (B −0.003, p = 0.003) and for spine L1-4 DAS28ESR3 (B −0.013, p = 0.037). Variables independently associated with change in bone density for the 2–10 years follow up period was for femoral neck menopause (B −0.042, p = 0.001) and smoking (B −0.034, p = 0.008), for total hip menopause (B −0.059, p < 0.001) and smoking (B −0.033, p = 0.011) and for spine L1-4 female gender (B −0.105, p < 0.001) and RF (B −0.056, p = 0.015).

## Discussion

In our prospective observational study of early RA patients the annual rate of bone loss was higher in the 0–2 year period compared with the 2–10 year period. This was however only seen in men and in premenopausal women and not in post-menopausal women. In the 0–2 year period disease and treatment variables were more frequently found to be associated with bone loss than well known risk factors for osteoporosis e.g. smoking and post-menopausal status, whereas the opposite was seen for the 2–10 years period.

A high rate of generalized bone loss in early RA patients has been reported from the pre-biologic era [5, 6]. In one study the annual rate of bone loss was reported to be −3.6% at femoral neck and −2.1% at lumbar spine [6] and in another study −1.7% at femoral neck and −2.7% at lumbar spine [5]. These figures are significantly higher than found in our study. This is most likely explained by the high proportion of patients treated with biologic DMARDs in our study. In the 0–2 year period 18.5% and in the 2–10 year period 62.6% were ever users of biologic DMARDs.

In the literature there is convincing evidence that treatment with TNF inhibitors reduces generalized bone loss in RA patients [9–11]. The bone protective effect of TNF inhibitors in RA patients may not only be explained by the strong anti-inflammatory properties of TNF inhibitors. Anti-TNF therapy has been shown to reduce radiographic joint damage and peri-articular bone loss in RA independent of clinical response [18, 19].

GCs are known as potent anti-inflammatory drugs and are frequently used in RA, however, they also cause osteoporosis [20, 21]. In RA this negative effect of GC on bone has been questioned. Studies have even reported BMD to increase in RA patients using GC [9, 14]. In our study neither ever use of prednisolone nor ever use of any GC was found to be associated with bone loss. Only for cumulative equivalent prednisolone doses a significant association in adjusted analysis was found to be associated with bone loss but only at total hip in the 0–2 year period. This must be interpreted with cautiousness as ever use of biologic DMARDs also was found to be significantly associated with bone loss at femoral neck in the first 2 years. This may be explained by that both the use of biologic treatment and GC in our observational study behaves as a surrogate marker for disease activity. It is important to emphasize that bone density data cannot directly be translated into reduced fracture risk in patients treated with GC because patients using GC may have a higher risk of fracture independent of the BMD level [22].

In our cohort of 92 RA patients with mean age 51 years at baseline one patient had an ankle and femur fracture and two other patients suffered clinical costa fracture during follow up. These numbers of new fractures seem to be low. In a 5 year follow up study of 102 female RA patients mean age 61 years with disease duration 17 years 16% of the patients had a new non vertebral fracture and 19% had a new vertebral fracture on spine X-ray during follow up [23]. In two studies using data from health care utilization databases, commercial insurance plan databases or administrative health care databases no association between use of biologic therapy and risk of fractures was shown [4, 24]. One hypothesis explaining this negative finding may be that patients treated with biologic drugs had a higher disease activity than patients not staring treatment with biologics, thus having a higher risk of fracture prior to treatment which then was reduced during treatment. In RA patients registered in the American CORRONA registry postmenopausal status, high mHAQ and prednisolone use was found to be associated with increased risk of fracture whereas the use of TNF alone but not in combination with methotrexate was reported to be associated with reduced fracture risk [25].

In our study we found no association between use of ART and rate of bone loss. ART however has been shown to reduce bone loss in RA [12]. Surprisingly in our male RA patients bone density at lumbar spine increased in the 2–10 years period. As we describe in the Results section this was explained by new bone formation seen more frequently in men than in women.

Our study does have limitations. One obvious limitation of this study is the missing baseline BMD data from a rather large proportion of included RA patients. The same pattern and range of change in BMD using imputation of missing data was however also seen when patients with available data for all four time points was analyzed. Thus we believe the missing BMD data at baseline is not a crucial limitation of our study. Lack of standardization of treatment also makes it difficult to explore for associations. However on the other hand our study reflects the bone density outcome in early RA patients aggressively treated in ordinary daily clinical practice in the new millennium. Unfortunately our study was not design to explore the prevalence and incidence of vertebral fractures.

The study design without having age and gender matched controls from the background population is another obvious limitation. Comparing our data with previous reports on bone loss in non-RA populations may indicate that bone loss in RA patients with more suppressed inflammation as seen in the 2–10 years period approaches the levels reported in the literature [26–29]. In a Finnish population the annual rate of BMD loss was estimated in cross sectional studies to be −0.2% at lumbar spine and −0.3% at femoral neck in men, [27] and in females above 39 years −0.7% and −1.3%, respectively [28]. However, in a population followed longitudinally annual percentage bone loss at total hip was reported to be 0.0% in men and −0.2% in women [29].

## Conclusions

In conclusion our study adds evidence that aggressive treatment of inflammation especially with biologic DMARDs in early RA reduces the rate of bone loss significantly. These findings are promising and encouraging as they indicate that modern aggressive treatment can reduce the burden of osteoporosis in RA patients. Future fracture studies however are needed to confirm if osteoporotic fractures in RA are reduced in the new millennium.

## Authors’ original submitted files for images

Below are the links to the authors’ original submitted files for images. Authors’ original file for figure 1 Authors’ original file for figure 2 Authors’ original file for figure 3
