# Supplementary figures and images for: Generalized bone loss in early rheumatoid arthritis patients followed for ten years in the biologic treatment era
Source: BMC Musculoskelet Disord. 2014 Sep 2;15:289. doi: 10.1186/1471-2474-15-289 (PMC4161846; doi:10.1186/1471-2474-15-289)

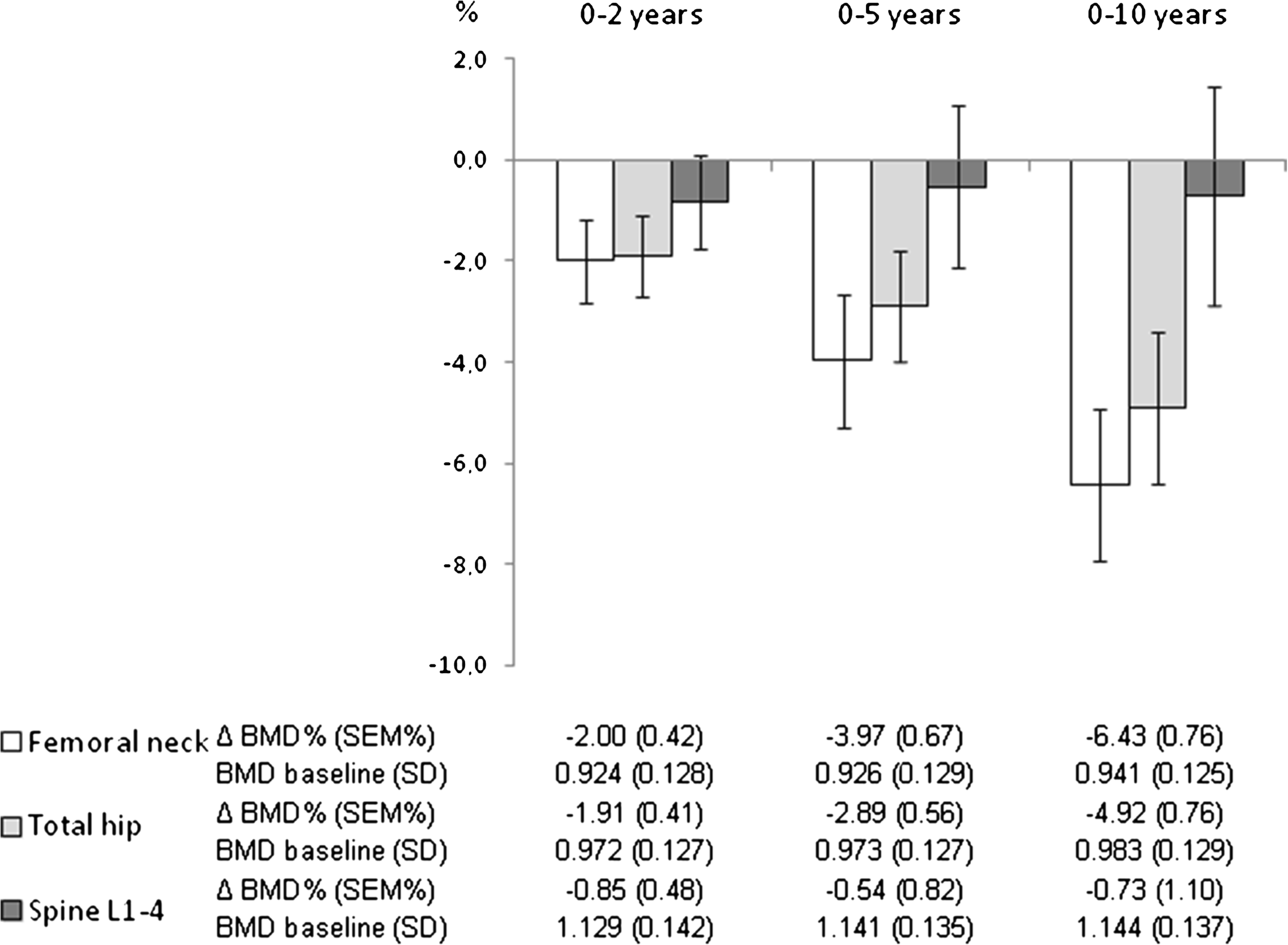

Supplement: Supplementary file 1 — Authors’ original file for figure 1 [file 12891_2014_2233_MOESM1_ESM.tif]

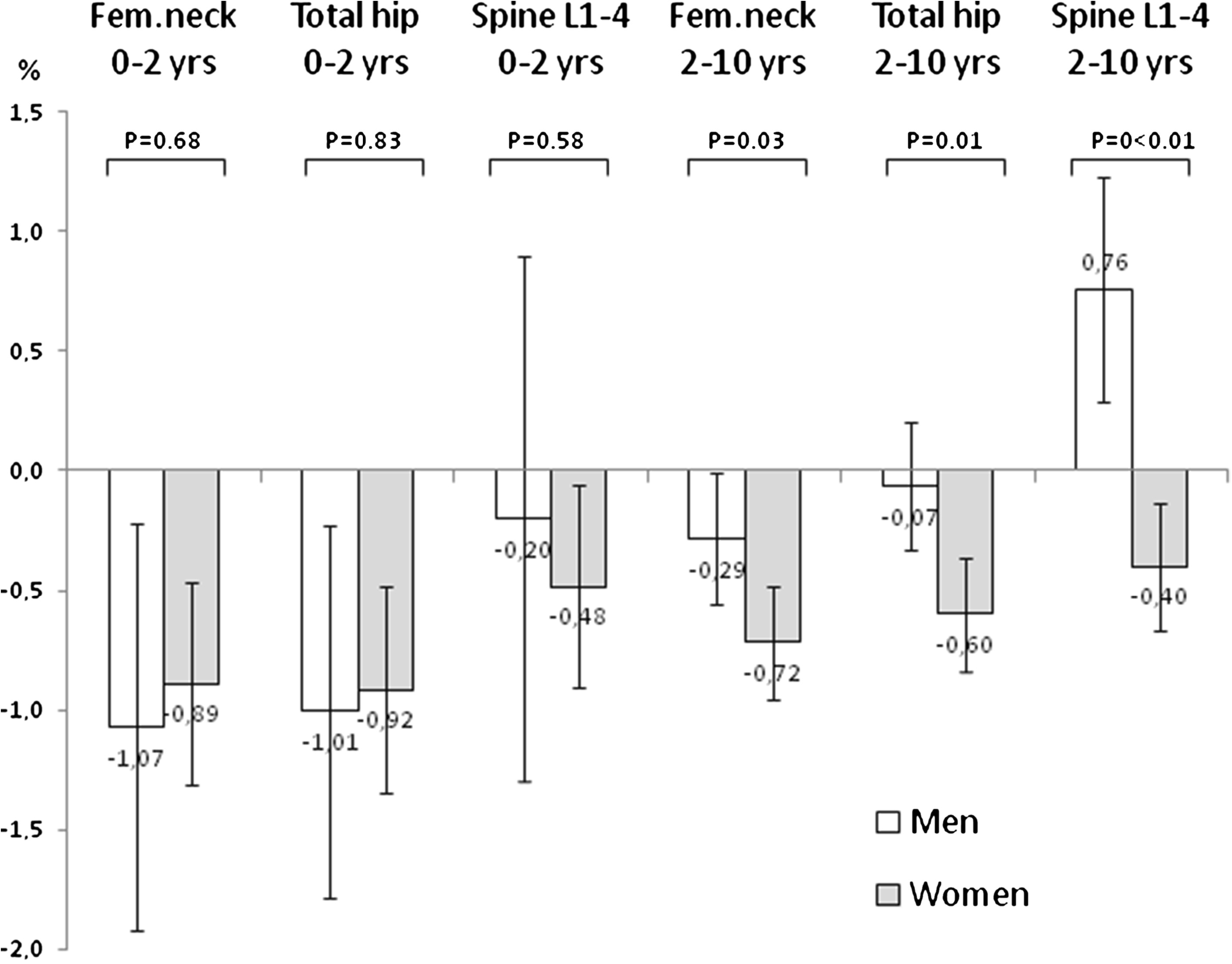

Supplement: Supplementary file 2 — Authors’ original file for figure 2 [file 12891_2014_2233_MOESM2_ESM.tif]

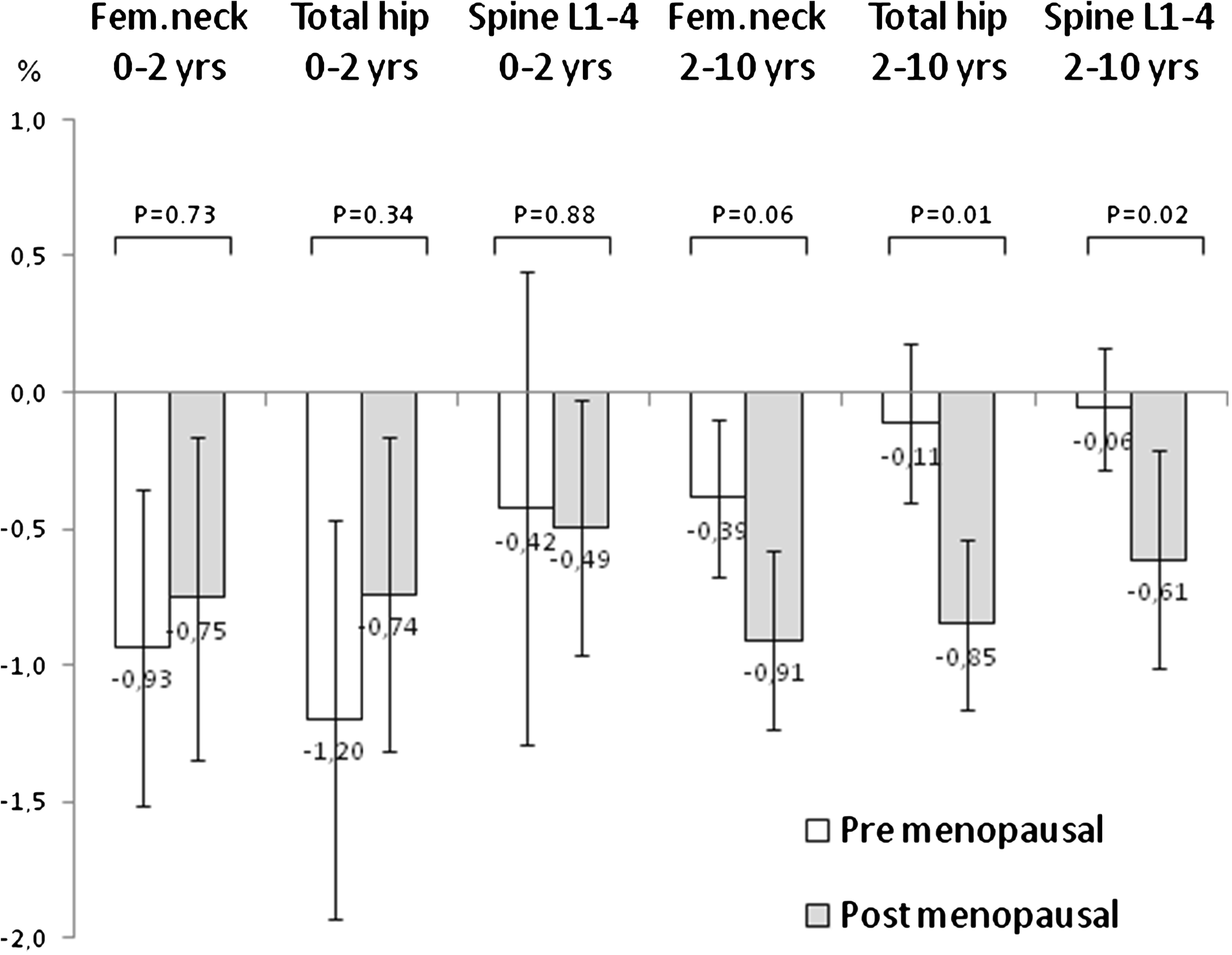

Supplement: Supplementary file 3 — Authors’ original file for figure 3 [file 12891_2014_2233_MOESM3_ESM.tif]
